# Discovery of an alternative pathway of peptidoglycan biosynthesis: A new target for pathway specific inhibitors

**DOI:** 10.1093/jimb/kuab038

**Published:** 2021-06-11

**Authors:** Yasushi Ogasawara, Tohru Dairi

**Affiliations:** Graduate School of Engineering, Hokkaido University, N13 & W8, Kita-ku, Sapporo, Hokkaido 060-8628, Japan; Graduate School of Engineering, Hokkaido University, N13 & W8, Kita-ku, Sapporo, Hokkaido 060-8628, Japan

**Keywords:** Peptidoglycan biosynthesis, Inhibitors, *Xanthomonas*

## Abstract

Peptidoglycan in bacterial cell walls is a biopolymer consisting of sugars and amino acids and plays important role in maintaining cell integrity from the environment. Its biosynthesis is a major target for antibiotics and the genes and enzymes involved in the biosynthetic pathway have been well studied. However, we recently identified an alternative pathway in the early stage of peptidoglycan biosynthesis in *Xanthomonas oryzae*, a plant pathogen causing bacterial blight disease of rice. The distribution of the alternative pathway is limited to relatively few bacterial genera that contain many pathogenic species, including *Xylella* and *Stenotrophomonas*, besides *Xanthomonas*. Thus, the alternative pathway is an attractive target for the development of narrow-spectrum antibiotics specific to pathogens. In this minireview, we summarize the discovery of the alternative pathway and identification of its specific inhibitors.

## Introduction

**Fig. 1.**
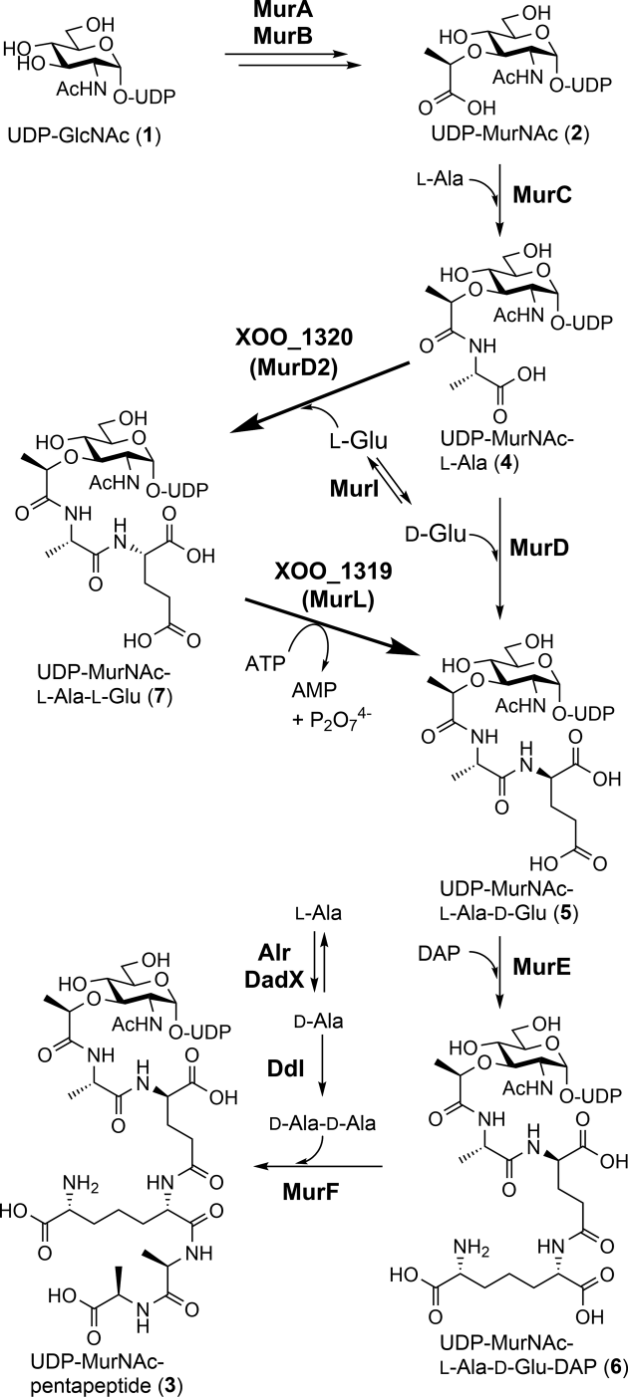
Early stage of peptidoglycan biosynthesis. The reactions in the alternative pathway are shown in bold arrow lines.

Peptidoglycan is a mesh-like macromolecule consisting of peptides and oligosaccharides that exists on the outside of the plasma membrane of most bacteria. The oligosaccharide chains are formed with two alternating sugar units, *N*-acetylglucosamine (GlcNAc) and *N*-acetylmuramic acid (MurNAc), and the oligopeptide chains on MurNAc that crosslink with each other to form a mesh-like rigid structure in the peptidoglycan. Because human cells do not have this structure, many antibiotics targeting peptidoglycan biosynthesis, such as penicillin, vancomycin, fosfomycin, liposidomycin, and CPZEN-45, have been discovered and developed to date (Bugg & Walsh, 1992; Muller et al., 2017; Osada, 2019). As illustrated in Fig. 1, the biosynthesis of peptidoglycan has been well characterized (Bouhss et al., 2008; El Zoeiby et al., 2003; van Heijenoort, 2001). UDP-GlcNAc (**1**), derived from fructose 6-phosphate, is first converted into UDP-MurNAc (**2**) by two enzymes, MurA and MurB. UDP-MurNAc then undergoes successive peptide chain elongation with l-alanine (Ala), d-glutamic acid (Glu), a diamino acid (l-lysine or meso-diaminopimelic acid, DAP), and d-Ala-d-Ala by four ATP-grasp enzymes (MurC, D, E, and F) to biosynthesize the key intermediate UDP-MurNAc-pentapeptide (**3**) via **4–6**. In this process, d-Glu is generally supplied by glutamate racemases (MurI) from l-Glu or by d-amino acid aminotransferases from α-ketoglutaric acid (Doublet et al., 1993; Doublet et al., 1992; Thorne et al., 1955). d-Ala-d-Ala is biosynthesized with PLP-dependent alanine racemases (Alr and DadX) followed by ATP-dependent d-Ala:d-Ala ligase (Ddl). The biosynthetic steps after this intermediate occur in a membrane-associated manner and are initiated by MurY catalyzing transfer of the MurNAc-pentapeptide moiety onto undecaprenyl phosphate (C_55_) to form the first lipid intermediate, known as lipid I (Boyle & Donachie, 1998; Chung et al., 2013). Subsequently, the glycosyltransferase MurG catalyzes addition of GlcNAc to lipid I, yielding lipid II (Crouvoisier et al., 1999). After the translocation of the GlcNAc-MurNAc-pentapeptide moiety of lipid II toward the periplasmic space by a flippase (MurJ), peptidoglycan glycosyltransferases (PGTs) catalyze the second last step in peptidoglycan biosynthesis and form β(1→4) glycosidic linkages between the disaccharide units. Finally, crosslink formation of the oligo peptide chains between the amino group of the diamino acid and the carboxyl group of d-Ala completes peptidoglycan biosynthesis.

We have been searching for alternative biosynthetic pathways of primary metabolites and previously discovered the futalosine pathway, an alternative pathway for menaquinone biosynthesis, operating in some bacteria including pathogenic *Helicobacter pylori* and *Campylobacter jejuni* (Arakawa et al., 2011; Dairi, 2012; Hiratsuka et al., 2008; Hiratsuka et al., 2009). Because menaquinone biosynthesis is indispensable for the survival of bacteria and some useful intestinal bacteria including lactobacilli use the well-studied canonical pathway, the futalosine pathway is a promising target for the development of specific drugs against *H. pylori* and *C. jejuni* and several inhibitors have been identified (Ogasawara & Dairi, 2019; Ogasawara et al., 2017; Shimizu et al., 2018; Tanaka et al., 2011). In addition, we recently discovered an alternative pathway in the early stage of peptidoglycan biosynthesis (Feng et al., 2017). Because the alternative pathway operates only in some bacteria including *Xanthomonas* and *Stenotrophomonas*—a plant pathogen and a hospital infection-causing bacterium, respectively—this pathway is an attractive target for the development of specific drugs against the abovementioned pathogens. In this minireview, we summarize the discovery of the alternative pathway of peptidoglycan biosynthesis and identification of pathway specific inhibitors.

## The Alternative Pathway Found in *Xanthomonas oryzae*

In the course of genome mining to search for alternative pathways of primary metabolite biosynthesis, we noticed that some bacteria such as the plant pathogen *X. oryzae* lacked orthologs of genes responsible for biosynthesis of d-Glu, including Glu racemases and d-amino acid aminotransferases. Because *X. oryzae* biosynthesizes peptidoglycan containing a d-Glu moiety in the structure, we speculated that an unprecedented enzyme is involved in the biosynthesis of d-Glu in *X. oryzae*. To identify the gene responsible for d-Glu formation in *X. oryzae*, we carried out functional complementation experiments of d-Glu auxotrophic *Escherichia coli* WM335 (Dougherty et al., 1993) with a genomic DNA library of *X. oryzae* MAFF311018. Consequently, we obtained many clones that grew without d-Glu supplementation and found that two genes, XOO_1319 and XOO_1320 (Ochiai et al., 2005), which were annotated as a protein with unknown function and MurD (UDP-MurNAc-l-Ala:d-Glu ligase), respectively, were required for the auxotroph complementation.

Considering the putative function of XOO_1320 based on homology analysis, we originally proposed that XOO_1319 is a novel Glu racemase. Accordingly, we incubated recombinant XOO_1319 and XOO_1320 with enzymatically prepared UDP-MurNAc-l-Ala (**4**) and l-Glu in the presence of ATP and Mg^2+^, and observed the formation of UDP-MurNAc-l-Ala-d-Glu (**5**) by LC-MS and chiral amino acid analysis. However, no Glu racemase activity was detected with recombinant XOO_1319 even though we tested reactions with various cofactors including pyridoxal-5ʹ-phosphate (PLP), a cofactor for typical amino acid racemases. In addition, XOO_1320 exhibited extremely low enzymatic activity compared with that of *E. coli* MurD when we used **4**, d-Glu and ATP as the substrates. Therefore, we next examined whether XOO_1320 used **4** and l-Glu instead of d-Glu as substrates. Surprisingly, LC-MS analysis revealed the formation of a new product that had the same mass spectrum but a different retention time compared with **5**. NMR and chiral analysis revealed that the product was UDP-MurNAc-l-Ala-l-Glu (**7**), indicating that XOO_1320 was a novel MurD-like enzyme that used l-Glu instead of d-Glu. We thus designated XOO_1320 as UDP-MurNAc-l-Ala-l-Glu synthetase, MurD2.

**Fig. 2.**
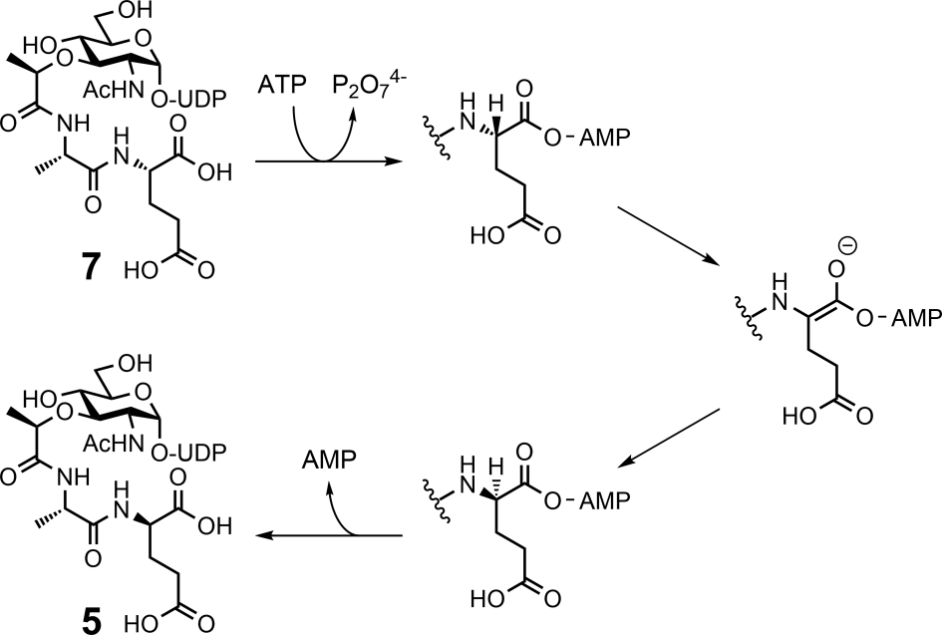
Proposed reaction mechanism of MurL (XOO_1319).

We next examined *in vitro* XOO_1319 reactions under various conditions and found that recombinant XOO_1319 efficiently converted **7** into UDP-MurNAc-l-Ala-d-Glu (**5**) in the presence of ATP and Mg^2+^. The reaction required one equivalent of ATP as a co-substrate and formed AMP. This is the first example of an epimerase that uses ATP as a co-substrate. Furthermore, a reaction with **5** and ATP did not yield **7**, indicating that epimerization by XOO_1319 is unidirectional from L to D. This feature contrasts with most epimerases, which yield an equilibrium mixture of the two epimers. Although XOO_1319 lacks known conserved domains and the reaction mechanism is not clear at this point, we hypothesized that the XOO_1319 reaction first activates the α-carboxylate group of **7** as the corresponding adenylate and subsequent epimerization and hydrolysis yields **5** (Fig. 2). ATP is likely important for unidirectional epimerization via substrate recognition in the adenylation step. We designated XOO_1319 as UDP-MurNAc-l-Ala-l-Glu epimerase, MurL.

Analysis of publicly available genome databases revealed that MurD2 and MurL are present in some genera of Gammaproteobacteria (*Xylella, Stenotrophomonas, Dyella, Frateuria, Rhodanobacter, Pseudoxanthomonas*, and *Lysobacter* besides *Xanthomonas*), Alphaproteobacteria (*Devosia, Pelagibacterium*, and *Parvularcula*), and rare actinobacteria (*Micromonospora, Actinoplanes, Verrucosispora*, and *Salinispora*). Because these bacteria lack a gene for D-Glu formation, MurD2/MurL orthologs are likely involved in the alternative peptidoglycan biosynthesis pathway. Among these taxa, the functions of MurD2/MurL orthologs found in *Micromonospora* sp. and *Salinispora tropica* were confirmed by *in vitro* assay (Feng et al., 2017).

## Stereospecific Substrate Recognition of MurD2

Although MurD2 exhibits moderate homology to canonical MurD, MurD2 recognizes l-Glu, an enantiomer of the MurD substrate, as the amine donor. MurD and MurD2 are members of the ATP-grasp superfamily enzymes, which catalyze amide bond formation from a carboxylic acid and an amine. In general, enzymes of the ATP-grasp superfamily first activate a substrate carboxylic acid at the expense of ATP to generate a reactive acylphosphate as an intermediate and subsequent nucleophilic attack by an amine of the acylphosphate completes the amide bond formation (Powers & Meister, 1976). Because MurD and MurD2 likely recognize Glu stereoisomers through a subtle difference in their Glu recognition sites, we next investigated how MurD2 recognizes l-Glu by homology modeling, docking simulation, and biochemical studies of *X. oryzae* MurD2. We first constructed homology models of the MurD2 structure based on crystal structures of *E. coli* MurD (MurD_ec_) with UDP-MurNAc-l-Ala, ADP and Mg^2+^ (representing the substrate binding state), and with UDP-MurNAc-l-Ala-d-Glu and Mg^2+^ (representing the product-binding state) (Feng et al., 2019). While the overall structure of MurD2 was well aligned with that of MurD_ec_, some amino acid residues in the Glu recognition site were different in the two enzymes. In particular, Asp182, Lys348, Ala414, and Leu416 located near the d-Glu in MurD_ec_ were replaced with Glu197, Arg358, Pro433, and Phe435, respectively, in the MurD2 model structure, suggesting that these four residues are important for the l-Glu recognition in MurD2. Thus, we performed site-directed mutagenesis analysis by substituting these residues in MurD2 with the corresponding residues in MurD_ec_ and examined both d-Glu- and l-Glu-ligase activities of the mutant enzymes. Although two MurD2 mutant enzymes (P433A and F435L) exhibited the same stereospecificity toward Glu substates as the wild-type MurD2, it was changed in the two mutants E197D and R358K. Furthermore, E197D/R358K double mutation completely switched the activity of MurD2 from UDP-MurNAc-l-Ala-l-Glu synthetase to the canonical UDP-MurNAc-l-Ala-d-Glu synthetase.

We next tried to switch MurD_ec_ to MurD2 by site-directed mutagenesis on the abovementioned amino acid residues. However, the mutant enzymes, including D182E/K348R, were unable to accept l-Glu. This observation was consistent with a previous report that the substrate specificity of MurD_ec_ was very strict for d-Glu. Similar site-directed mutagenesis experiments were also carried out using MurD of *Streptococcus mutans* (MurD_sm_), because it has an Arg residue at the position corresponding to Arg358 of MurD2 and *in vitro* experiments showed that the recombinant MurD_sm_ slightly accepted l-Glu besides its physiological substrate d-Glu. However, all mutant enzymes still accepted d-Glu. Therefore, we performed random mutagenesis to obtain a mutant enzyme of MurD_sm_ that accepts l-Glu. After preparation of a plasmid library of MurD_sm_ mutants by error-prone PCR, we performed functional complementation experiments on *E. coli* WM335 with XOO_1319 and mutated MurD_sm_ in the same manner as mentioned above. As a result, we identified MurD_sm__K330T as the sole clone and showed that MurD_sm__K330T slightly improved substrate acceptability of l-Glu compared with the wild-type enzyme. The corresponding mutant enzyme of MurD_ec_ (K319T) also accepted l-Glu, which is the first example of a change to the chiral specificity of MurD_ec._ In the model structure, K330 in MurD_sm_ and K319 in MurD_ec_ are located near the ADP molecule and likely play an important role in controlling the substrate and product specificities of the MurD reaction. Taking these results together, we found that a few amino acid residues in MurD/MurD2 control their chiral specificity.

## Exploration of Specific Inhibitors

**Fig. 3.**
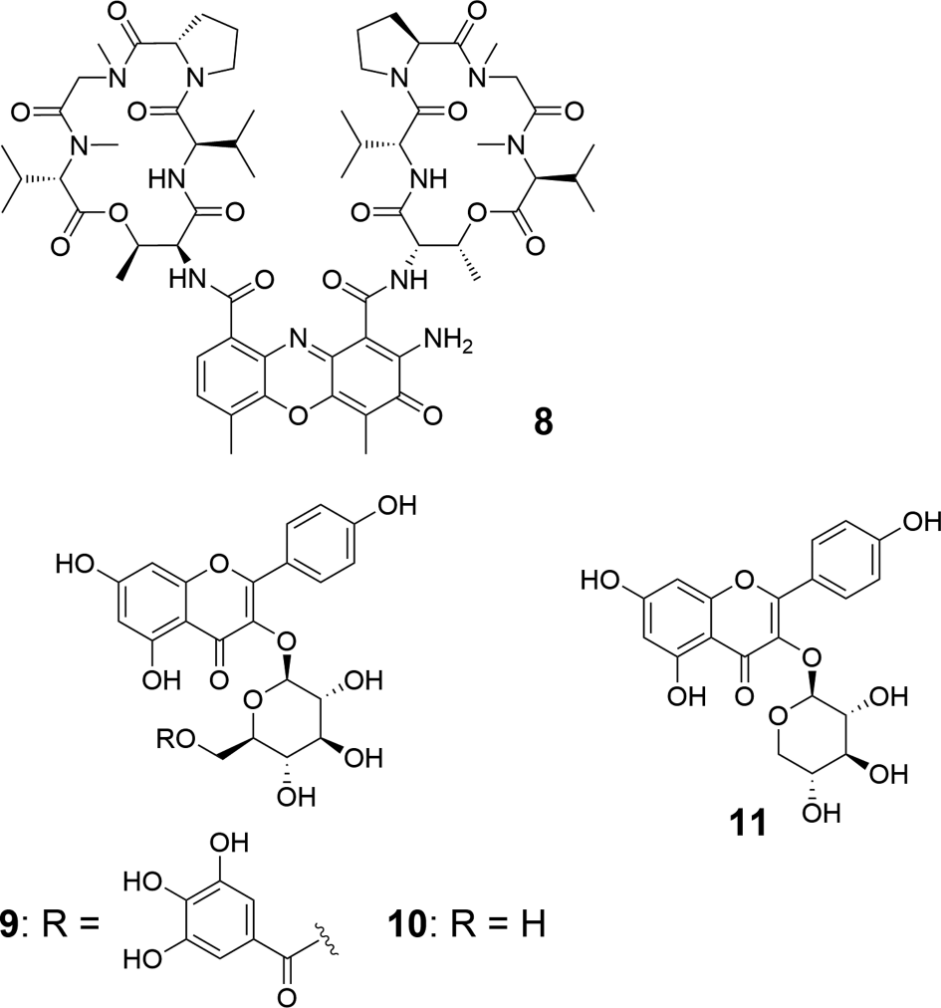
Structures of specific inhibitors of the alternative pathway.

As mentioned above, the alternative pathway is specific to some pathogenic bacteria such as *Xanthomonas* and *Stenotrophomonas*. Thus, we searched for inhibitors of the alternative pathway from a culture broth library of actinomycetes to develop specific drugs against these pathogens. We first developed a paper disk-agar diffusion assay using two actinobacterial strains, *Micromonospora* sp. ATCC 39149 and *Streptomyces lividans* TK23, as test microorganisms. Although the two strains are similar in terms of their 16S rDNA sequence, genome size, and G + C content, the former uses the alternative pathway and the latter uses the canonical pathway. A compound that inhibits the enzymes in the alternative pathway would specifically repress the growth of *Micromonospora* sp. We screened approximately 200 actinomycete culture broths and found that a culture broth of *Streptomyces parvulus* NBRC 13193 specifically inhibited the growth of *Micromonospora* sp. (Ogasawara et al., 2020). The sample also showed growth inhibitory activity against *X. oryzae*. Accordingly, we purified the active compounds by HPLC using a C-18 column and obtained compound **8** as a red solid. By NMR, high-resolution (HR)-MS, and single crystal x-ray analysis, the structure of **8** was confirmed to be actinomycin D, a known antibiotic with anticancer activity (Fig. 3) (Dalgliesh & Todd, 1949; Kirk, 1960; Waksman et al., 1946). The minimum inhibitory concentration (MIC) against *X. oryzae* was 25 μM, while the MIC values against other Gram-negative bacteria (*E. coli, Pseudomonas putida*, and *Pseudomonas syringae*) were > 1 mM. *In vitro* inhibition assay with recombinant enzymes of *X. oryzae* revealed that **8** inhibited the MurD2 reaction but not that of MurL, and the apparent inhibition constants (*K*_i_ values) of **8** for **5**, l-Glu, and ATP were 0.46, 1.9, and 2.8 mM, respectively.

Recently, Morita et al. searched for inhibitors of the alternative pathway from a library of Myanmar medicinal plants and found that a 70% ethanol extract of *Woodfordia fruticosa* (Lythraceae) flowers inhibited the reaction of a MurD2 ortholog in *Stenotrophomonas maltophilia*, SmltD (Lee et al., 2021). This plant is used as traditional herbal medicine in East Asia and known to be rich in flavonoids and tannins (Das et al., 2007). By purification and structural elucidation of the active compounds, three known natural products, kaempferol 3-*O*-(6′′-galloyl)-β-d-glucopyranoside (**9**) (Braca et al., 2003), astragalin (**10**) (Lavoie et al., 2017), and Juglalin (**11**) (Lavoie et al., 2017) were identified by NMR and HR-MS (Fig. 3). Detailed kinetic analysis showed that **9** is most potent (IC_50_ 0.46 mM), followed in order by **10** (1.2 mM) and **11** (2.1 mM). In addition, **9** was shown to be a competitive inhibitor of ATP with an apparent *K*_i_ value of 0.11 mM, suggesting that these compounds bind to the ATP-binding site in SmltD. Docking studies using a model structure of SmltD also supported this binding mode. The galloyl moiety of **9** occupies the adenosine-binding sites and the remaining part binds to the cleft between the N-terminal domain and the central domain of SmltD. The weaker inhibitory activities of **10** and **11** compared with **9** are consistent with the lack of recognition of the galloyl moiety by SmltD.

## Conclusion

In this minireview, we summarized the discovery of the alternative pathway of peptidoglycan biosynthesis and exploration of its specific inhibitors. The pathway involves a MurD-like ATP grasp family enzyme (MurD2) that attaches l-Glu instead of d-Glu to UDP-MurNAc-l-Ala and a novel ATP-dependent epimerase (MurL). Comparison of MurD2 with MurD_ec_ revealed that only a few amino acid residues in MurD/MurD2 are important to control the chiral-specific recognition of the Glu substrate and we successfully switched the activity of MurD2 to the canonical UDP-MurNAc-l-Ala-d-Glu synthetase by substitution of two amino acids. We also altered the chiral specificity of MurD_ec_ by a single amino acid mutation. Conversely, MurL is an unprecedented peptide epimerase that catalyzes unidirectional epimerization from UDP-MurNAc-l-Ala-l-Glu to UDP-MurNAc-l-Ala-d-Glu using ATP as a co-substrate. Because the alternative pathway operates in some pathogenic bacteria and is an attractive target for the development of narrow-spectrum antibiotics specific to pathogens, we also searched for inhibitors of MurD2/MurL. To date, we have identified four compounds that inhibit the MurD2 reaction, although these showed low affinities. Further screening of MurD2/MurL inhibitors with narrow-spectrum antibiotics, which have recently attracted increasing attention because of their low propensity for bacterial multiple drug resistance, is necessary.
